# Correlation of histological components with tumor invasion in pulmonary adenocarcinoma

**DOI:** 10.1186/1477-7819-12-388

**Published:** 2014-12-17

**Authors:** Youngkyu Moon, Kyung Soo Kim, Sook Whan Sung, Kyo-Young Lee, Young Kyoon Kim, Jin Hyoung Kang, Yeon Sil Kim, Jae Kil Park

**Affiliations:** Department of Thoracic & Cardiovascular Surgery, The Catholic University of Korea, Seoul St. Mary’s Hospital, Seoul, Republic of Korea; Department of Hospital Pathology, The Catholic University of Korea, Seoul St. Mary’s Hospital, Seoul, Republic of Korea; Department of Internal Medicine, The Catholic University of Korea, Seoul St. Mary’s Hospital, Seoul, Republic of Korea; Department of Radiation Oncology, The Catholic University of Korea, Seoul St. Mary’s Hospital, Seoul, Republic of Korea

**Keywords:** Adenocarcinoma component, Lung cancer, Acinar, Papillary, Micropapillary, Solid, Lepidic

## Abstract

**Background:**

Pulmonary adenocarcinoma (PA) is the most common histologic type of primary lung cancer. Generally, adenocarcinoma was composed by five major components. The present study aimed to evaluate changes in the composition of adenocarcinoma components as the tumor grows; in addition, to analyze the correlation between the occupancy rates of histologic components of the tumor in regard to prognosis.

**Methods:**

Pathologic data were retrospectively evaluated for 206 patients who underwent curative resection of PA. We investigated how histologic component occupancy rates changed as tumor size and N stage increased. To evaluate local invasiveness, the major components of the present group and absent group of pleural invasion, lymphatic invasion, and vascular invasion were compared.

**Results:**

The mean percentages of acinar and solid components significantly increased with an increase in size (*P* = 0.006, *P* < 0.001) ; however, the percentage of lepidic components decreased (*P* < 0.001). In cases with a solid component and a micropapillary component, a gradual increase was found with an increase N stage (*P* = 0.001, *P* < 0.001); however the percentage of lepidic components decreased (*P* < 0.001). Average differences of histologic components dependent upon whether pleural, lympathic and vascular invasion were present, the difference of micropapillary and lepidic components were statistically significant. With logistic regression analysis, as the occupancy rate of the lepidic component increased, the probability of pleural invasion, lymphatic invasion, and vascular invasion decreased; in cases with a micropapillary component, as the occupancy rate of increased, the probability of lymphatic invasion and vascular invasion increased. In multivariate analysis using the Cox propotional hazards model, the occupancy rates of acinar(p = 0.043; odds ratio = 1.023), micropapillary(p = 0.002; odds ratio = 1.051) and lepidic (p = 0.005; odds ratio = 0.966) components were significantly associated with recurrence.

**Conclusions:**

The lower the occupancy rate of a lepidic component and the higher the occupancy rates of acinar, solid, and micropapillary components, the likelihood of tumor progression increased. In addition, as the occupancy rate of a lepidic component decreased and a micropapillary component increased, local invasiveness and recurrence rate increased; thus, increasing the probability of a poor prognosis.

## Background

Pulmonary adenocarcinoma (PA) is the most common histologic type of primary lung cancer [1]. Since Noguchi et al. reported a histopathologic study of primary pulmonary adenocarcinoma located in the peripheral lung, in which the tumor size was < 2 cm in diameter [2], significant attention has been paid to the histologic classification of PA. In 2011, the subtypes of adenocarcinoma were newly proposed by the International Association for the Study of Lung Cancer (IASLC), the American Thoracic Society (ATS), and the European Respiratory Society (ERS); the significance of histologic classification was reemphasized in this revision. According to this classification, the components of adenocarcinoma were classified into five major histologic components, depending on the growth pattern or shape of tumor: acinar, papillary, micropapillary, solid, and lepidic. The percentage of each histologic component was recorded in 5% increments; the subtype of the adenocarcinoma was accordingly determined by the occupancy rate. For the classification, since the five types of components are mixed in most invasive adenocarcinomas, the descriptor “predominant”, which indicated the highest occupancy component, was affixed to subtype labels [3].

The majority of primary adenocarcinomas that occur in the peripheral lung have the radiologic appearance of a ground glass opacity nodule (GGN) at the incipient stage; in most cases, the lesion becomes more solid as the tumor grows [4, 5]. Moreover, GGN lesions are primarily lepidic; as the size increased, this lepidic component gradually decreases and is replaced with acinar or papillary components. This type of change in the composition of cells in the tumor is considered to be a specific feature solely of PA; to date, the change in the tumor composition during growth has not been fully evaluated.

The most important prognostic factor of non-small cell lung cancer is its anatomical stage; this staging is commonly determined by the American Joint Committee on Cancer (AJCC) TNM stage [6]. However, since many cases with the same staging have different prognoses, it is difficult to determine an accurate prognosis solely by stage. Particularly because histologic components are mixed in lung adenocarcinomas, it should be considered that various tumor characteristics and prognostic factors are dependent upon the occupancy rate. Therefore, even though histologic analyses of adenocarcinomas have been conducted in the past, most of the studies were focused on a prognosis analysis of the five subtypes determined by their predominant component [7, 8]. Furthermore, studies that conducted a prognosis analysis dependent upon the occupancy rates of tumor components are lacking. In view of this situation, we conducted a study to evaluate changes in the composition of adenocarcinoma components as the tumor grows; in addition, we analyzed the correlation between the occupancy rates of histologic components of the tumor in regard to prognosis.

## Methods

### Patients

From March 2011 through September 2013 at Seoul St. Mary’s Hospital in Korea, 407 patients were diagnosed with non-small cell lung cancer and underwent pulmonary resection that entailed more than a lobectomy for an attempt to achieve a radical cure. Among these 407 patients, 275 were diagnosed with adenocarcinoma. The following patients were excluded from the evaluation: treatment with neoadjuvant chemotherapy preoperatively; diagnosed with atypical adenomatous hyperplasia (AAH), adenocarcinoma in situ (AIS), or minimally invasive adenocarcinoma (MIA); had multiple lesions, staging was not possible because a lymph node dissection was not performed; or the occupancy rates of histologic components were not recorded. Ultimately, 206 patients were included in this retrospective chart review. This study was approved by the Institutional Review Board of Seoul St. Mary's Hospital (The Catholic University of Korea).

### Histologic evaluation

The five components of adenocarcinoma were recorded in 5% increments. The recording method was followed via the recommendations of IASLC/ATS/ERS [3]. If the component was not typed as acinar, papillary, micropapillary, solid or lepidic, it was classified as “other”. The seventh edition of the American Joint Committee on Cancer(AJCC) TNM classification was applied [6]. To evaluate local invasiveness, the major components of the present group and absent group of pleural invasion, lymphatic invasion, and vascular invasion were compared. All pathologic evaluations were made by a board-certified pathologist.

### Statistics

The averages of the respective component occupancy rates in each tumor group classified by size and N stage were compared by ANOVA and the Kruskal Wallis H test; the averages of the respective component occupancy rates of each component classified with pleural invasion, lymphatic invasion, and vascular invasion were comparatively analyzed via the t-test. In addition, logistic regression was used for the analysis of the factors influencing pleural invasion, lymphatic invasion, and vascular invasion. Statistical significance was set at *P* < 0.05.

## Results

### Patient characteristics

The mean patient age was 63.13 years (range: 33–85 years). A total of 91 patients (44.2%) were men, and 115 patients (55.8%) were women. In regard to the location of tumor onset, it was central in 15 cases (7.3%) and peripheral in 191 cases (92.7%); thus, the tumor onset was primarily in the peripheral area. In regard to stage, it was IA in 114 cases (55.3%), IB in 36 cases (17.5%), IIA in 22 cases (10.7%), IIB in 9 cases (4.4%), IIIA in 21 cases (10.2%), IIIB in 1 case (0.5%), and IV in 3 cases (1.5%).

Classification by tumor size was in accordance with T stage criteria; the tumor size was < 2 cm in 80 cases (38.9%), ≥ 2 cm and < 3 cm in 65 cases (31.6%), ≥ 3 cm and < 5 cm in 8 cases (3.8%), and ≥ 7 cm in 2 cases (0.9%). In regard to N stage, it was N0 in 164 cases (79.6%), N1 in 22 cases (10.7%), and N2 in 20 cases (9.7%). Pleural invasion was present in 50 cases (24.3%), lymphatic invasion was present in 95 cases (46.1%), and vascular invasion was present in 40 cases (19.4%) (Table 1).

**Table 1:** **Patient characteristics**

		n	%
Sex	Male	91	44.2
	Female	115	55.8
Location	Central	15	7.3
	Peripheral	191	92.7
Stage	IA	114	55.3
	IB	36	17.5
	IIA	22	10.7
	IIB	9	4.4
	IIIA	21	10.2
	IIIB	1	0.5
	IV	3	1.5
Tumor size	<2 cm	80	38.9
	≥2 cm and <3 cm	65	31.6
	≥3 cm and <5 cm	51	24.8
	≥5 cm and <7 cm	8	3.8
	≥7 cm	2	0.9
N stage	N0	164	79.6
	N1	22	10.7
	N2	20	9.7
Pleural invasion	Absent	156	75.7
	Present	50	24.3
Lymphatic invasion	Absent	111	53.9
	Present	95	46.1
Vascular invasion	Absent	166	80.6
	Present	40	19.4

### Average occupancy rates of histologic components

The average occupancy rate of each component among the 206 patients was: acinar, 39.6%; lepidic, 33.1%; papillary, 11.3%; solid, 8.8%; micropapillary, 4.5%; and other, 2.6%.

**Figure 1:**
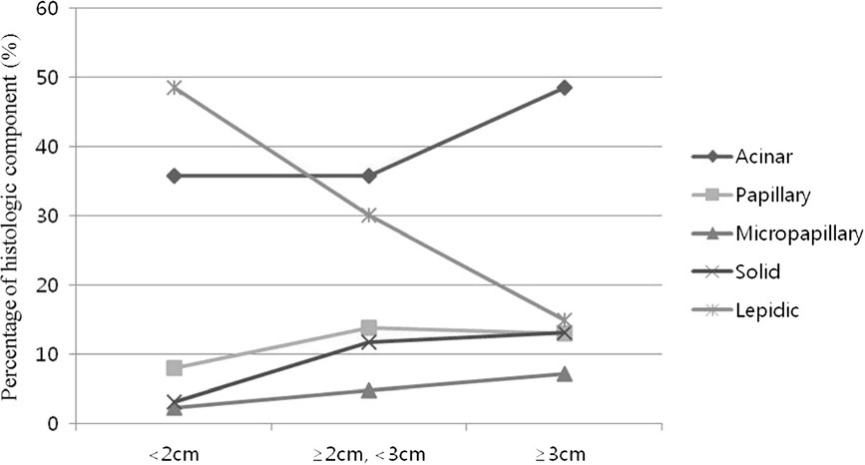
**Sizes of each histologic component.** The mean percentages of acinar (*P* = 0.006) and solid (*P* < 0.001) components significantly increased with an increase in size; however, the percentage of lepidic components decreased (*P* < 0.001).

### Size and percentages of histologic components

Even though the patients were classified into five groups, based on tumor size and in accordance with T stage criteria, the number of cases in the group of ≥ 5 cm and < 7 cm was only 8, and the number of cases in the group ≥ 7 cm was only 2; therefore we regrouped the patients into three groups: < 2 cm, ≥ 2 cm and < 3 cm, and ≥3 cm; the number of cases in each group was: < 2 cm, 80 (38.8%); ≥ 2 cm and < 3 cm, 65 (31.6%), and ≥ 3 cm, 61 (29.6%). We compared the change in the average histologic component occupancy rate in accordance with the increase of size. In cases of a predominant acinar component, when the size was < 2 cm, it was 35.6%; it was 35.8% when the size was ≥ 2 cm and < 3 cm. However it increased significantly to 48.52% when the size was ≥ 3 cm (*P* = 0.006). In cases with a solid component, it also increased as the size increased: < 2 cm, 3.1%; ≥ 2 cm and < 3 cm, 11.8%; and ≥ 3 cm, 13.1% (*P* < 0.001). However, in cases with a lepidic component, it decreased as the size increased: < 2 cm, 49.6%; ≥ 2 cm and < 3 cm, 30.0%; and ≥ 3 cm, 14.9%. In micropapillary component cases, it increased as the size increased: < 2 cm, 2.3%; ≥ 2 cm and < 3 cm, 4.8%; and ≥ 3 cm, 7.1%; however, the difference was not statistically significant (*P* = 0.143). In cases with a papillary component, there was no significant change (*P* = 0.138) (Figure 1).

**Figure 2:**
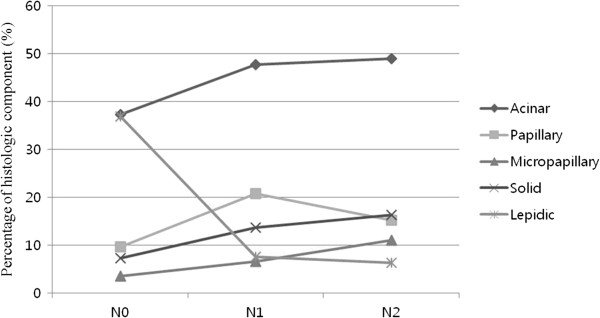
**N stage and percentage of each histologic component.** The mean percentages of solid (*P* = 0.001) and micropapillary (*P* < 0.001) components significantly increased with increasing size; however, the percentage of lepidic components decreased.

### N stage and percentages of histologic components

N stage was classified as N0, N1, and N2 groups in accordance with TNM classification, and the differences in the average histologic component occupancy rates were compared accordingly. In cases with a solid component, the average histologic component occupancy rates were: N0, 7.3%; N1, 13.6%; and N2, 16.3%; a gradual increase was found (*P* = 0.001). In cases with a micropapillary component, a gradual increase was also found: N0, 3.5%; N1, 6.6%; and N2, 11.0% (*P* < 0.001). However in cases with a lepidic component, a gradual decrease was found: N0, 39.9%; N1, 7.5%; and N2, 6.3% (*P* < 0.001); no significant changes were found in cases with an acinar (*P* = 0.051) or papillary component (*P* = 0.053) (Figure 2).

**Figure 3:**
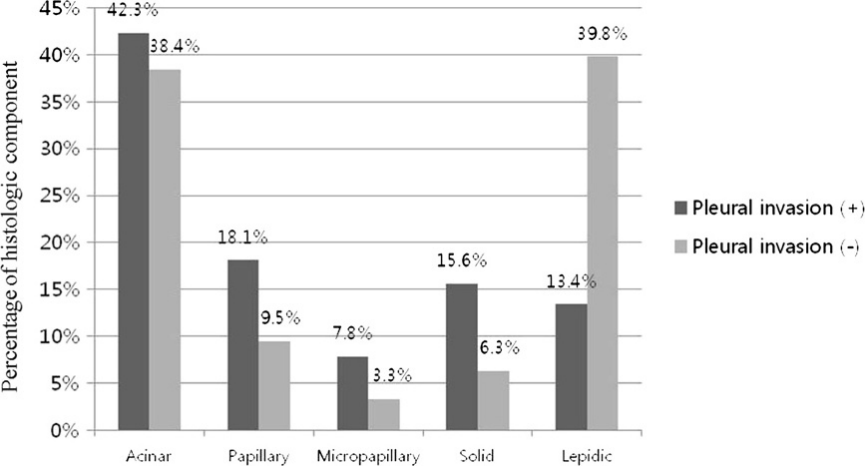
**Average differences of histologic components dependent on whether pleural invasion was present.** The differences for papillary (*P* = 0.015), micropapillary *P* < 0.001), solid (*P* < 0.001), and lepidic (*P* < 0.001) were statistically significant.

### Local invasiveness

#### Pleural invasion

The average histologic component occupancy rates were compared between the groups with and without pleural invasion. In cases with a papillary component, it was 9.5%, in cases with a micropapillary component, it was 3.3%, and in cases with a solid component, it was 6.3% in the group without pleural invasion; a significant increase was found in the group with pleural invasion: papillary component, 18.1% (*P* = 0.015); micropapillary component, 7.8% (*P* < 0.001)*;* and solid component, 15.6% *(P* < 0.001). In cases with a lepidic component, it was 39.8% in the group without pleural invasion and 13.4% in the group with pleural invasion; thus, showing a statistically significant lowering-aspect (*P* < 0.001) (Figure 3).

**Figure 4:**
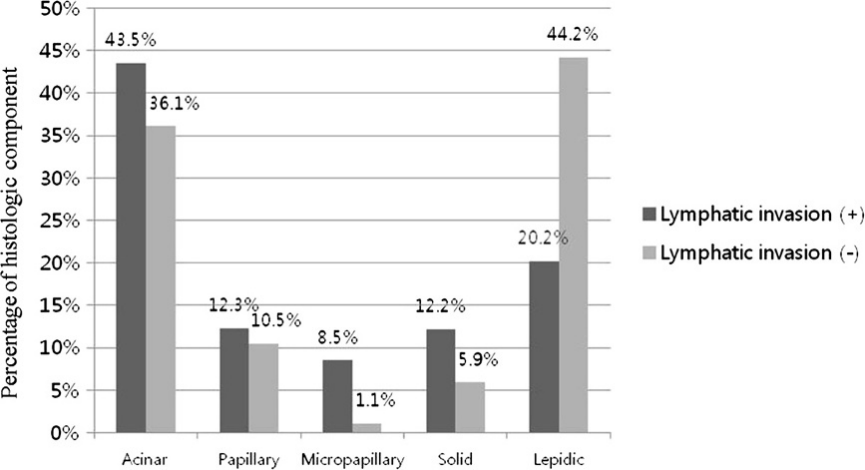
**Average differences of histologic components dependent upon whether lymphatic invasion was present.** The differences for micropapillary (*P* < 0.001), solid (*P* < 0.001), and lepidic (*P* < 0.001) components were statistically significant.

#### Lymphatic invasion

The average histologic component occupancy rates were also compared between the groups with and without lymphatic invasion. In the group without lymphatic invasion, the following was found: cases with a micropapillary component, 1.1%; and cases with a solid component, 5.9%. In the group with lymphatic invasion, a significant increase was found: cases with a micropapillary component, 8.5% (*P* < 0.001); and cases with a solid component, 12.2% (*P* < 0.001). In cases with a lepidic component, it was 44.2% in the group without lymphatic invasion and 20.2% in the group with lymphatic invasion; thus, showing a statistically significant lowering aspect (*P* < 0.001) (Figure 4).

**Figure 5:**
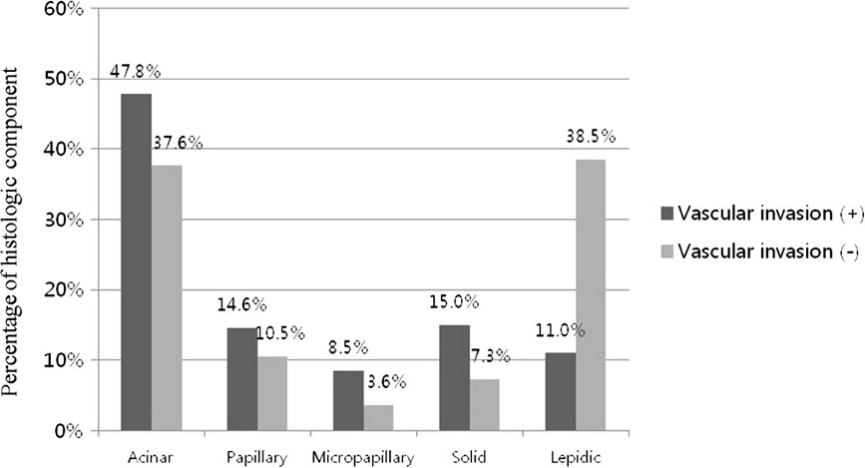
**Average differences of histologic components dependent upon whether vascular invasion was present.** The differences of acinar (*P* = 0.028), micropapillary (*P* = 0.002), solid (*P* = 0.027, and lepidic (*P* < 0.001) components were statistically significant.

#### Vascular invasion

The average histologic component occupancy rates were also compared between the groups with and without vascular invasion. Among the group without vascular invasion, in cases with a acinar component, it was 37.6%, in cases with a micropapillary component, it was 3.6%, and in cases with a solid component, it was 8.5%. Among the group with vascular invasion, a significant increase was found: in cases with a acinar component, it was 47.8% (*P* = 0.028), in cases with a micropapillary component, it was 8.5% (*P* = 0.002), and in cases with a solid component, it was 15.0% (*P =* 0.027). In cases with a lepidic component, it was 38.5% in the group without vascular invasion and 11.0% in the group with vascular invasion; thus, showing a statistically significant lowering-aspect (*P* < 0.001) (Figure 5). These findings were similar to those of the cases with a lymphatic component.

#### Logistic regression analysis

Multivariate logistic regression analysis was conducted with the covariates of age, sex, location of tumor, and size of tumor; this was done to evaluate the influence of the increase or decrease of the occupancy rate of histologic components on pleural invasion, lymphatic invasion, and vascular invasion.

In cases with pleural invasion, the possibility of invasion increased as the occupancy rates of papillary components (*P* = 0.022; odds ratio = 1.018 (1.003-1.034)) and solid components increased (*P* = 0.040; odds ratio = 1.018 (1.001-1.036); a similar decrease was found as the occupancy rate of the lepidic increased (*P* < 0.001; odds ratio = 0.965 (0.948-0.983). In cases of lymphatic invasion and vascular invasion, only the micropapillary and lepidic types showed significant results. As the percentage of the micropapillary component increased, the likelihood of lymphatic invasion (*P* < 0.001; odds ratio = 1.134 (1.058-1.216) and vascular invasion (*P* = 0.047; odds ratio = 1.027 (1.000-1.054)) increased. As the percentage of the lepidic component increased, the likelihood of lymphatic invasion (*P* < 0.001; odds ratio = 0.979 (0.968-0.990)) and vascular invasion (*P* =0.001; odds ratio = 0.965 (0.944-0.968)) decreased (Table 2).

**Table 2:** **Multivariate analysis of histologic components for local invasiveness by logistic regression model**

	Histologic components	Odds ratio	95% Confidence interval	***P***Value
Pleural invasion	Acinar	1.004	0.990-1.018	0.561
	Papillary	1.018	1.003-1.034	0.002
	Micropapillary	1.025	0.998-1.053	0.070
	Solid	1.018	1.001-1.036	0.040
	Lepidic	0.965	0.948-0.983	<0.001
Lymphatic invasion	Acinar	1.008	0.996-1.019	0.193
	Papillary	1.003	0.988-1.017	0.724
	Micropapillary	1.134	1.058-1.216	<0.001
	Solid	1.012	0.996-1.029	0.149
	Lepidic	0.979	0.968-0.990	<0.001
Vascular invasion	Acinar	1.013	0.997-1.028	0.101
	Papillary	1.007	0.989-1.024	0.458
	Micropapillary	1.027	1.000-1.054	0.047
	Solid	1.010	0.992-1.028	0.279
	Lepidic	0.965	0.944-0.986	0.001

### Recurrence

**Figure 6:**
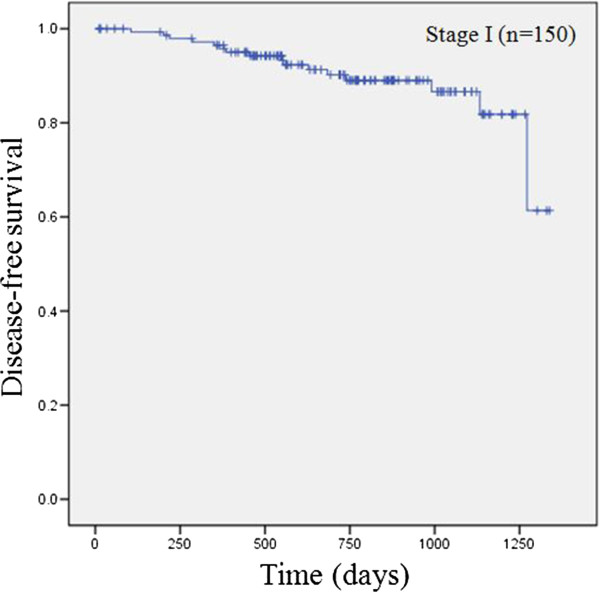
**Disease free survival in stage I patients.**

Follow-up was performed for all patients. The median follow-up period was 735.9 days(range, 12 to 1338 days). During follow up, 35 patients (17%) of all patients (n = 206) and 16 patients of stage I patients (n = 150) experienced recurrence. In stage I, 3-year disease free survival was 86.6% (Figure 6). Multivariate analysis were performed to identify independent risk factors of recurrence using the Cox propotional hazards model. Covariate factors were age, sex, location of tumor, and size of tumor. The occupancy rates of acinar(p = 0.043; odds ratio = 1.023), micropapillary(p = 0.002; odds ratio = 1.051) and lepidic (p = 0.005; odds ratio = 0.966) components were significantly associated with recurrence (Table 3).

**Table 3:** **Multivariate analysis of disease-free survival**

Histologic components	Odds ratio	95% Confidence interval	***P***Value
Acinar	1.023	1.001-1.045	0.043
Papillary	1.001	0.974-1.028	0.960
Micropapillary	1.051	1.019-1.085	0.002
Solid	1.003	0.973-1.034	0.840
Lepidic	0.966	0.943-0.990	0.005

## Discussion

Adenocarcinoma of the lung has various subtypes and it is considered that diversified subtypes will have different prognoses. Following the proposal of a new histological classification of adenocarcinoma by IASLC/ATS/ERS in 2011, studies on its utility have been steadily published. Up to the present, some data were presented that histologic subtype is related to prognosis [9]. According to Yoshizawa et al.’s analysis of the prognosis of 514 stage I adenocarcinoma patients, lepidic predominant adenocarcinoma showed the best prognosis and solid predominant, as well as micropapillary predominant adenocarcinomas, showed a poor prognosis [10]. In cased where curative surgery was performed regardless of stage, Tsuta et al. analyzed the prognosis of adenocarcinoma subtypes and reported the following 5-year survival rates: lepidic, 93%; acinar, 67%; papillary, 74%; micropapillary, 62%; and solid-predominant, 58% [8]. Darin et al. reported the following 5-year survival rates: papillary-predominant, 80%; lepidic-predominant, 71%; micropapillary-predominant, 55%; acinar-predominant, 43%; and solid-predominant, 39%. However both studies reported no statistically significant differences in the survival rate [7]. Despite the probability of a significant difference dependent on the predominant component, no definitive data has been presented. This may be because even though adenocarcinoma subtypes are classified by their predominant components, more than 90% of adenocarcinomas fall into the category of a “mixed type”, consisting of diverse cell types [2, 10, 11]. Thus, the components other than the predominant component might influence the prognosis. Studies up to the present have focused on the analysis of the predominant component. With this study, we investigated the extent of influence of the occupancy rate of each component on the progression and prognosis of the tumor.

Tumor progression can be defined by tumor size and lymph node metastases, in accordance with the TNM stage. In this study we investigated how histologic component occupancy rates changed as tumor size increased. It was confirmed with statistical significance that as tumor size increased, the rates of acinar and solid components increased, and the rate of lepidic component decreased. In cases with a micropapillary component, the rate of increase accelerated as tumor size increased; however, the rate of increase was not statistically significant. Cases with lymph node metastases exhibited similar results, and we found that the rates of solid and micropapillary components increased and the rate of lepidic component decreased with statistical significance as the N stage of lymph node metastases increased (N0, N1, and N2). In cases with an acinar component, the growth rate increased as the N stage increased; however, the increase did not reach statistical significance. In summary, we found that as the tumor progressed, the occupancy rate of the lepidic component decreased while that of the solid component increased.

Pleural invasion, lymphatic invasion, and vascular invasion are known prognostic factors of lung cancer. Cases with pleural invasion are considered to be one of factors influencing prognosis because pleural invasion is one of the factors included in the TNM stage [12]. However, since pleural invasion is significantly influenced by the location and size of tumor, controversy exists regarding its role as a prognostic factor, especially in stage I cancer [13]. In cases with lymphatic invasion and/or vascular invasion, there are many reports that suggest that the aforementioned factors have an influence on prognosis [11, 14–18]. In this study, we comparatively evaluated the differences in the occupancy rates of histologic components in the two groups with and without pleural invasion, lymphatic invasion, and vascular invasion; we used logistic regression analysis to confirm the correlation between the rate and the degree of invasion. To evaluate the influence of the change in the occupancy rate of histologic component on invasion, multivariate logistic regression analysis was conducted with the covariates of age, sex, location of tumor, and size of tumor. By first evaluating the mean rate comparison, the groups with pleural invasion, lymphatic invasion, or vascular invasion all were found to have higher mean occupancy rates of micropapillary and solid components; in addition, they all were found to have lower occupancy rates of a lepidic component, compared with the group without those invasions. With logistic regression analysis, as the occupancy rate of the lepidic component increased, the probability of pleural invasion, lymphatic invasion, and vascular invasion decreased; in cases with a micropapillary component, as the occupancy rate of increased, the probability of lymphatic invasion and vascular invasion increased. In summary, these results suggested that the occupancy rates of the lepidic component and the micropapillary component were related to local invasion; thus, an increase of the lepidic component occupancy rate appears to be a good prognostic factor, while an increase of the micropapillary component occupancy appears to be a poor prognostic factor. The presence of a micropapillary component has been suggested to be a poor prognostic factor and the literature contains reports that the recurrence rate increased in the presence of a micropapillary component [19–21]. However, no detailed studies are available on an increase of occupancy rate of micropapillary component and its influence on prognosis. In this regard, it appears that our study has significance in that a poor prognosis can be predicted in accordance with an increase of the occupancy rate.

Our data were relatively recent data, so we couldn’t analyzed 5-year overall survival. However, we performed 3-year disease free survival analysis. In multivariate analysis, an increase of micropapillary component occupancy and an decrease of lepidic component occupancy were significantly associated with recurrence. This results showed that the occupancy rates of micropapillary and lepidic components are related with prognosis.

All of the foregoing results contributed somewhat to the prediction of the characteristics and changes of the typical histologic components of pulmonary adenocarcinoma. Each component would have different characteristics, which could influence the clinical course. If the occupancy rates of these histologic components were analyzed in detail and the characteristics in accordance with the change in the occupancy rates were revealed, individualization of a treatment protocol for adenocarcinoma could defined; thus, helping to maximize the benefits of the treatment.

Our study had several limitations. It is retrospective and single center. The patient sample was relatively small; this particularly impacted categorization by tumor size. Even though the classification was made in compliance with T stage criteria of TNM stage, the number of patients in the group > 5 cm was only 10; thus, the group was combined with the group ≥ 3 cm and < 5 cm. If the patient sample was larger, more size subdivisions could be made and more accurate results could have been attained; furthermore, this would increase the significance of the study. Long-term follow-up was not conducted. Long-term follow-up regarding the influence of each histologic component could provide a more detailed analysis of the recurrence rate and mortality.

## Conclusions

In most cases, pulmonary adenocarcinoma consists of various histologic components and the occupancy rate of each component changes as the tumor progresses. The lower the occupancy rate of a lepidic component and the higher the occupancy rates of acinar, solid, and micropapillary components, the likelihood of tumor progression increased. In addition, as the occupancy rate of a lepidic component decreased and the occupancy rate of a micropapillary component increased, local invasiveness and recurrence rate increased; thus, increasing the probability of a poor prognosis.

## References

[1] Parkin DM, Ferlay J, Curado MP, Bray F, Edwards B, Shin HR, Forman D (2010). Fifty years of cancer incidence: CI5 I-IX. Int J Cancer.

[2] Noguchi M, Morikawa A, Kawasaki M, Matsuno Y, Yamada T, Hirohashi S, Kondo H, Shimosato Y (1995). Small adenocarcinoma of the lung. Histologic characteristics and prognosis. Cancer.

[3] Travis WD, Brambilla E, Noguchi M, Nicholson AG, Geisinger KR, Yatabe Y, Beer DG, Powell CA, Riely GJ, Van Schil PE, Garg K, Austin JHM, Asamura H, Rusch VW, Hirsch FR, Scagliotti G, Mitsudomi T, Huber RM, Ishikawa Y, Jett J, Sanchez-Cespedes M, Sculier JP, Takahashi T, Tsuboi T, Vansteenkiste J, Wistuba I, Yang PC, Aberle D, Brambilla C, Flieder D (2011). International association for the study of lung cancer/american thoracic society/european respiratory society international multidisciplinary classification of lung adenocarcinoma. J Thorac Oncol.

[4] Asamura H, Suzuki K, Watanabe S, Matsuno Y, Maeshima A, Tsuchiya R (2003). A clinicopathological study of resected subcentimeter lung cancers: a favorable prognosis for ground glass opacity lesions. Ann Thorac Surg.

[5] Higashiyama M, Kodama K, Yokouchi H, Takami K, Mano M, Kido S, Kuriyama K (1999). Prognostic value of bronchiolo-alveolar carcinoma component of small lung adenocarcinoma. Ann Thorac Surg.

[6] Stephen BD, Carolyn R, Apri C, Frederick G, L Andy T (2010). AJCC Cancer Staging Manual.

[7] Westaway DD, Toon CW, Farzin M, Sioson L, Watson N, Brady PW, Marshman D, Mathur MM, Gill AJ (2013). The International Association for the Study of Lung Cancer/American Thoracic Society/European Respiratory Society grading system has limited prognostic significance in advanced resected pulmonary adenocarcinoma. Pathology.

[8] Tsuta K, Kawago M, Inoue E, Yoshida A, Takahashi F, Sakurai H, Watanabe S, Takeuchi M, Furuta K, Asamura H, Tsuda H (2013). The utility of the proposed IASLC/ATS/ERS lung adenocarcinoma subtypes for disease prognosis and correlation of driver gene alterations. Lung Cancer.

[9] Sica G, Yoshizawa A, Sima CS, Azzoli CG, Downey RJ, Rusch VW, Travis WD, Moreira AL (2010). A grading system of lung adenocarcinomas based on histologic pattern is predictive of disease recurrence in stage I tumors. Am J Surg Pathol.

[10] Yoshizawa A, Motoi N, Riely GJ, Sima CS, Gerald WL, Kris MG, Park BJ, Rusch VW, Travis WD (2011). Impact of proposed IASLC/ATS/ERS classification of lung adenocarcinoma: prognostic subgroups and implications for further revision of staging based on analysis of 514 stage I cases. Mod Pathol.

[11] Motoi N, Szoke J, Riely GJ, Seshan VE, Kris MG, Rusch VW, Gerald WL, Travis WD (2004). Lung adenocarcinoma: modification of the 2004 WHO mixed subtype to include the major histologic subtype suggests correlations between papillary and micropapillary adenocarcinoma subtypes, EGFR mutations and gene expression analysis. Am J Surg Pathol.

[12] Yoshida J, Nagai K, Asamura H, Goya T, Koshiishi Y, Sohara Y, Eguchi K, Mori M, Nakanishi Y, Tsuchiya R, Miyaoka E (2009). Visceral pleura invasion impact on non-small cell lung cancer patient survival: its implications for the forthcoming TNM staging based on a large-scale nation-wide database. J Thorac Oncol.

[13] David E, Thall PF, Kalhor N, Hofstetter WL, Rice DC, Roth JA, Swisher SG, Walsh GL, Vaporciyan AA, Wei C, Mehran RJ (2013). Visceral pleural invasion is not predictive of survival in patients with lung cancer and smaller tumor size. Ann Thorac Surg.

[14] Nentwich MF, Bohn BA, Uzunoglu FG, Reeh M, Quaas A, Grob TJ, Perez D, Kutup A, Bockhorn M, Izbicki JR, Vashist YK (2013). Lymphatic invasion predicts survival in patients with early node-negative non-small cell lung cancer. J Thorac Cardiovasc Surg.

[15] Hanagiri T, Takenaka M, Oka S, Shigematsu Y, Nagata Y, Shimokawa H, Uramoto H, Yamada S, Tanaka F (2011). Prognostic significance of lymphovascular invasion for patients with stage I non-small cell lung cancer. Eur Surg Res.

[16] Gabor S, Renner H, Popper H, Anegg U, Sankin O, Matzi V, Lindenmann J, Smolle Juttner FM (2004). Invasion of blood vessels as significant prognostic factor in radically resected T1-3N0M0 non-small-cell lung cancer. Eur J Cardiothorac Surg.

[17] Ruffini E, Asioli S, Filosso PL, Buffoni L, Bruna MC, Mossetti C, Solidoro P, Oliaro A (2011). Significance of the presence of microscopic vascular invasion after complete resection of Stage I-II pT1-T2N0 non-small cell lung cancer and its relation with T-Size categories: did the 2009 7th edition of the TNM staging system miss something?. J Thorac Oncol.

[18] Tsuchiya T, Hashizume S, Akamine S, Muraoka M, Honda S, Tsuji K, Urabe S, Hayashi T, Yamasaki N, Nagayasu T (2007). UPstaging by vessel invasion improves the pathology staging system of non-small cell lung cancer*. Chest.

[19] Watanabe M, Yokose T, Tetsukan W, Imai K, Tsuboi M, Ito H, Ishikawa Y, Yamada K, Nakayama H, Fujino S (2013). Micropapillary components in a lung adenocarcinoma predict stump recurrence 8 years after resection: a case report. Lung Cancer.

[20] Sumiyoshi S, Yoshizawa A, Sonobe M, Kobayashi M, Fujimoto M, Tsuruyama T, Date H, Haga H (2013). Pulmonary adenocarcinomas with micropapillary component significantly correlate with recurrence, but can be well controlled with EGFR tyrosine kinase inhibitors in the early stages. Lung Cancer.

[21] Nitadori J, Bograd AJ, Kadota K, Sima CS, Rizk NP, Morales EA, Rusch VW, Travis WD, Adusumilli PS (2013). Impact of Micropapillary Histologic Subtype in Selecting Limited Resection vs Lobectomy for Lung Adenocarcinoma of 2 cm or Smaller. J Natl Cancer Inst.

